# Narrow band imaging versus lugol chromoendoscopy to diagnose squamous cell carcinoma of the esophagus: a systematic review and meta-analysis

**DOI:** 10.1186/s12885-016-3011-9

**Published:** 2017-01-13

**Authors:** Flavio Hiroshi Ananias Morita, Wanderley Marques Bernardo, Edson Ide, Rodrigo Silva Paula Rocha, Julio Cesar Martins Aquino, Mauricio Kazuyoshi Minata, Kendi Yamazaki, Sergio Barbosa Marques, Paulo Sakai, Eduardo Guimarães Hourneaux de Moura

**Affiliations:** 1 Gastrointestinal Endoscopy at University of Sao Paulo, Rua Capote Valente n 671, Pinheiros, São Paulo Zipcode 05409-002 Brazil; 2 University of Sao Paulo, Rua Maria Vidal 124, Perdizes, São Paulo, SP CEP 01253-040 Brazil; 3 Gastrointestinal Endoscopy at University of Sao Paulo, Rua Cristiano Viana 647 apto 141, Pinheiros, São Paulo, SP CEP 05411-001 Brazil; 4 Gastrointestinal Endoscopy at University of Sao Paulo, Alameda Ministro Rocha Azevedo 373, Cerqueira Cesar, São Paulo, SP CEP 01410-001 Brazil; 5 Gastrointestinal Endoscopy at University of Sao Paulo, Rua Teodoro Sampaio 498 apto 33, Pinheiros, São Paulo, SP CEP 05405-000 Brazil; 6 Gastrointestinal Endoscopy at University of Sao Paulo, Rua Cardoso de Almeida 840, Perdizes, São Paulo, SP CEP 05013-001 Brazil; 7 Gastrointestinal Endoscopy at University of Sao Paulo, Rua Malebranche 99 apto 142, Chácara Klabin, São Paulo, SP CEP 04116-160 Brazil; 8 Gastrointestinal Endoscopy at University of Sao Paulo, Avenida Libero Badoro 451, Bairro Jardim São Caetano, São Caetano do Sul, SP CEP 09581-610 Brazil; 9 Gastrointestinal Endoscopy at University of Sao Paulo, Rua Sincinato Braga 3712, Paraiso, São Paulo, SP CEP 01323-011 Brazil; 10 Gastrointestinal Endoscopy at University of Sao Paulo, Avenida Dr. Enéas de Carvalho Aguiar 255, sexto andar, bloco 3, Pinheiros, São Paulo, SP CEP 05403-000 Brazil

**Keywords:** Narrow band imaging, Lugol chromoendoscopy, Esophageal scquamous cell carcinoma, Esophageal neoplasm

## Abstract

**Background:**

In the early stage esophageal cancer, changes in the mucosa are subtle and pass unnoticed in endoscopic examinations using white light. To increase sensitivity, chromoscopy with Lugol’s solution has been used. Technological advancements have led to the emergence of virtual methods of endoscopic chromoscopy, including narrow band imaging (NBI). NBI enhances the relief of the mucosa and the underlying vascular pattern, providing greater convenience without the risks inherent to the use of vital dye. The purpose of this systematic review and meta-analysis was to evaluate the ability of NBI to diagnose squamous cell carcinoma of the esophagus and to compare it to chromoscopy with Lugol’s solution.

**Methods:**

This systematic review included all studies comparing the diagnostic accuracy of NBI and Lugol chromoendoscopy performed to identify high-grade dysplasia and/or squamous cell carcinoma in the esophagus. In the meta-analysis, we calculated and demonstrated sensitivity, specificity, and positive and negative likelihood values in forest plots. We also determined summary receiver operating characteristic (sROC) curves and estimates of the areas under the curves for both per-patient and per-lesion analysis.

**Results:**

The initial search identified 7079 articles. Of these, 18 studies were included in the systematic review and 12 were used in the meta-analysis, for a total of 1911 patients. In per-patient and per-lesion analysis, the sensitivity, specificity, and positive and negative likelihood values for Lugol chromoendoscopy were 92% and 98, 82 and 37%, 5.42 and 1.4, and 0.13 and 0.39, respectively, and for NBI were 88 and 94%, 88 and 65%, 8.32 and 2.62, and 0.16 and 0.12, respectively. There was a statistically significant difference in only specificity values, in which case NBI was superior to Lugol chromoendoscopy in both analyses. In the per-patient analysis, the area under the sROC curve for Lugol chromoendoscopy was 0.9559. In the case of NBI, this value was 0.9611; in the per-lesion analysis, this number was 0.9685 and 0.9587, respectively.

**Conclusions:**

NBI was adequate in evaluating the esophagus in order to diagnose high-grade dysplasia and squamous cell carcinoma. In the differentiation of those disorders from other esophageal mucosa alterations, the NBI was shown to be superior than Lugol.

## Background

Esophageal cancer is the eighth most common cancer in the world (4.9% of all cases). It is the sixth leading cause of death from cancer, causing 3.2% of deaths [1, 2]. In 2012, the estimated worldwide incidence was 455,800, with a mortality rate of 400,200 [3, 4]. The two main histological types are squamous cell carcinoma and adenocarcinoma [3]. In the areas with the greatest risk, which span from northern Iran through central Asia to the central-northern China (known as the “esophageal cancer belt”), 90% of cases are squamous cell carcinoma; squamous cell carcinoma is also the principal histological type worldwide [2].

The importance of individual risk factors in the development of squamous cell carcinoma of the esophagus varies by geographic region [3, 4]. Principal among the risk factors are consumption of alcohol and tobacco, with the same “field of cancerization” leading to squamous carcinomas of the head, neck, and lungs. Other risk factors are caustic esophageal stenosis, previous radiation therapy, achalasia, nutritional deficiencies (mainly zinc and selenium), low fruit and vegetable intake, diets high in N-nitroso compounds and red meat, diets low in folate, low socioeconomic status, poor oral hygiene, and ingestion of hot liquids [2–4].

Esophageal cancer is a highly aggressive disease, with a mortality rate of 88% [1]. Overall 5-year survival between 2002 and 2008 was estimated to be 16.9% [2]. Although survival rates are increasing, they remain low [2]. This is because most cases are diagnosed when the disease is in advanced stages [2]. One reason for late diagnosis is the aggressiveness of the disease: the cancer quickly invades the submucosa and affects regional lymph nodes at an early stage, since the lymphatics are located in the lamina propria of the esophagus, in contrast to the rest of the gastrointestinal tract, where they are located below the muscularis mucosa [4]. Another important reason is that the early lesions are asymptomatic and changes in the mucosa are subtle, which easily go unnoticed during endoscopic examination [2, 4]. Distant metastasis to the liver, bones, and lungs is found in approximately 30% of patients, and in this group, the average 5-year survival rate is 3.4% [2, 4]. This rate goes up to 37.8% in patients receiving diagnosis when the disease is restricted to the esophagus, which occurs in 22% cases [2].

Upper gastrointestinal endoscopy combined with biopsy is the method of choice for the diagnosis of squamous cell carcinoma of the esophagus. Technological advancements have brought an improvement in image quality, making it easier to identify not only easily visualized advanced neoplasms but also subtle changes that may correspond to early stages of the disease. Even with the substantial improvement in image quality, it is not always easy to identify these lesions. They may be faint reddish changes and irregularities in the mucosal relief, which can easily pass unnoticed in examination with white light. Esophageal chromoscopy with Lugol’s solution has been shown to increase the sensitivity of the lesion detection [5, 6].

In addition to improvements in image quality and magnification, technology has evolved to allow endoscopy with chromoscopy using virtual methods. As with conventional chromoscopy, these methods aim to enhance the surface mucosa and the underlying capillary pattern in the endoscopic examination, but do not require the application of dye to the esophagus. Principal among the advantages of virtual methods of endoscopic chromoscopy is practicality: they involve simply pressing a button, they do not cause chest discomfort (which is common when using Lugol’s solution), they bear no risk of pulmonary aspiration of the dye or allergic reaction, and they provide a safe assessment of the upper third of the esophagus. Disadvantages include the high cost of the device, which decreases accessibility. Ishihara et al. demonstrated lower sensitivity of narrow band imaging (NBI) when compared to chromoscopy with Lugol’s solution among inexperienced endoscopists, with an increase over the course of the study. For experienced endoscopists, sensitivity levels were consistent between the two methods [7]. Yokoyama et al. showed that NBI without magnification is suitable for esophageal assessment when searching for a neoplasm in a high-risk population, even without high-definition imagery [8].

Virtual chromoscopy methods have been studied and compared to white light and conventional chromoscopy in terms of diagnostic accuracy. The purpose of this systematic review and meta-analysis is to evaluate the ability of NBI to diagnose squamous cell carcinoma of the esophagus and to compare the results to those of chromoscopy with Lugol’s iodine solution. We use pathological results of biopsies or of dried sections as the gold standard.

This systematic review is necessary because publications comparing these two methods have found varying results. Considering the fact that NBI has the advantage of not using vital dye, consistent accuracy would be desirable for the use of NBI in clinical practice. We will investigate this variation in an attempt to find the best current evidence.

## Methods

### Protocol and registration

A protocol was established and documented prior to the beginning of the study in order to specify the eligibility criteria and methods of analysis for the studies included in this systematic review and meta-analysis. This protocol can be accessed at http://www.crd.york.ac.uk/PROSPERO. Its registration number is CRD42016037008.

### Eligibility criteria

#### Types of studies

This systematic review included all studies from which data extraction was possible in order to calculate the diagnostic accuracy of NBI and Lugol chromoendoscopy in esophageal assessment for the identification of high-grade dysplasia and/or squamous cell carcinoma, regardless of the primary outcome defined by the author. The meta-analysis used only studies which directly or indirectly supplied all the data necessary to calculate the sensitivity, specificity, and positive and negative likelihood values by patient and/or by lesion, which are analyzed separately in the current review. No abstracts or data from unpublished research were accepted. There were no restrictions in terms of language or date of publication.

#### Types of participants

There were no restrictions as to sex, age, risk factors, or previous diagnosis of squamous cell carcinoma of the esophagus in the study participants. According to the results, a subgroup analysis was performed if necessary.

#### Types of intervention

The intervention studied was upper GI endoscopy conducted by experienced endoscopists, in which the esophagus was assessed using NBI and Lugol chromoendoscopy in order to identify high-grade dysplasia and/or squamous cell carcinoma of the esophagus. We included studies that used and did not use magnification findings, and neither the device diameter (conventional or ultra-thin) nor the concentration of Lugol’s solution were taken into account. The gold standard for comparing the two methods was the results of pathological analysis of the biopsies or dried sections of the suspicious lesions identified by both methods.

#### Types of outcome measures

The primary outcome was the diagnostic accuracy testing for NBI and Lugol chromoendoscopy in identifying high-grade dysplasia and/or squamous cell carcinoma of the esophagus.

### Sources of information

In order to find articles, searches were conducted in virtual databases. There were no restrictions as to language. The databases used in the searches were PubMed/Medline (all years), Scopus (1988-present), Cochrane Central Register of Randomised Controlled Trials/CENTRAL (all years), LILACS (all years), and Cinahl (all years). The date of the last study in all the databases was 11/25/2015.

### Search

The search strategies that were utilized varied depending on the database and are specified below:

- PubMed/Medline: (esophagus OR esophageal) AND (neoplasms OR cancer OR squamous cell carcinoma OR dysplasia OR dysplastic) AND (narrow band imaging OR optical imaging OR nbi OR chromoendoscopy OR lugol OR iodine OR virtual imaging OR fice OR flexible spectral imaging color enhancement OR i-scan OR bli OR blue laser imaging OR endoscopy OR endoscopic) AND (diagnosis/broad[filter]);
- LILACS and Cochrane/CENTRAL: Esophageal cancer AND (narrow band imaging OR NBI);
- Scopus: (esophagus OR esophageal) AND (neoplasms OR cancer OR squamous cell carcinoma OR dysplasia OR dysplastic) AND (narrow band imaging OR optical imaging OR nbi OR chromoendoscopy OR lugol OR iodine OR virtual imaging OR fice OR flexible spectral imaging color enhancement OR i-scan OR bli OR blue laser imaging OR endoscopy OR endoscopic);
- Cinahl: (Esophageal cancer or esophageal Squamous Cell carcinoma) and (narrow band imaging or nbi).

### Study selection

The articles were initially selected after an assessment of the titles and abstracts in order to assess the relevancy of the full text. This process was carried out by two independent reviewers, and differences were resolved after a discussion and consensus with the participation of all of the authors of the current review.

The meta-analysis excluded retrospective studies, as well as studies in which the examinations were performed by inexperienced endoscopists or when low-grade dysplasia was considered as the target lesion (true positive), since it is not possible to distinguish these from high-grade dysplasias or squamous cell carcinoma for data collection. Also excluded were data from studies aimed at evaluating other outcomes that did not contain at least one patient evaluated using Lugol chromoendoscopy and NBI.

### Data collection process

The data were collected from the absolute numbers that were directly provided or were inferred through the information reported in the text. These were placed into 2 × 2 tables and separated for analysis by patient and/or by lesion according to the data that could be extracted from each article. These tables separated the true positives, false positives, true negatives, and false negatives. The meta-analysis only included studies that provided all the information necessary to completely fill in the table for at least one kind of analysis. Those which provided all of the data for analysis in the two subgroups were included in both. The entire process was completed by two independent authors and revised by all authors. Differences were resolved after a discussion and consensus among the authors.

### Data items

The criteria considered for the positivity of the methods in the meta-analysis were the same as those established by the authors, as long as they were suitable. We considered the presence of brownish areas (BAs) in evaluation with NBI to be fundamental, regardless of the magnification findings. In cases of Lugol chromoendoscopy, lesions measuring 5 mm or more in size that were not stained by iodine were considered fundamental, regardless of pink-color sign.

The criteria that could be used for lesions to be subjected to histopathological evaluation were the positive results using Lugol chromoendoscopy and/or NBI, or the positivity of only Lugol chromoendoscopy. Evident lesions of the esophagus (in other words, lesions that were easily identified by any method) were either considered or not considered for calculation in the meta-analysis depending on whether they were excluded by the author of the study in question.

### Risk of bias in individual studies

The quality of the studies included in the meta-analysis was assessed by two independent reviewers based on the pre-defined criteria and discussed with the entire group. We used the revised version of the Quality Assessment of Diagnostic Accuracy Studies (QUADAS-2) as a tool in this process. The QUADAS-2 criteria were used to assess the risks of biases and applicability in patient selection, in the methods in which the esophageal chromoscopies were conducted and interpreted, the way in which the lesions were classified using anatomical pathology, and their clinical significance. The risk of bias in the flow and time interval in which the tests were performed was also assessed.

The risk of bias in patient selection was also considered, and case-control or retrospective studies were excluded beforehand. We considered prospective and randomized studies with homogeneity between the two groups to be suitable, as well as cross-sectional studies in which both methods were evaluated in the same patient. Studies that excluded subtle lesions that were difficult to identify were considered inadequate with high risk of bias. Applicability was considered low when the selected patients were not at high risk for squamous cell carcinoma of the esophagus or when diagnosis of the disease had already been established without being previously cured.

When the endoscopist knew the pathology results of the recent esophageal biopsy or Lugol chromoendoscopy before conducting NBI, the study was considered to be unsuitable in the risk of bias assessment in the index test. With respect to the applicability of the methods, we evaluated whether a standardization of a well-established, suitable criteria was used to consider a lesion positive. For a lesion to be considered positive in NBI, BAs between the criteria were necessary. In Lugol chromoendoscopy the negative-iodine area was required to be greater than or equal to 5 mm.

With regard to the risk of bias and the applicability of the gold standard test, the pathologist had to be blinded to the method that was positive, showing the lesion and permitting its biopsy. Studies which considered low-grade dysplasias to be true positives, which provided sufficient data to distinguish them from high-grade dysplasia and squamous cell carcinomas, were included in the meta-analysis, and this separation was made in the calculations. These studies were considered to be at increased risk for biases and to have low applicability, since they did not differentiate lesions requiring intervention from those requiring monitoring.

To assess the risk of biases in flow and time, we considered cross-sectional studies in which NBI and Lugol chromoendoscopy were performed sequentially in the same procedure to be suitable. In prospective studies, patients needed to be randomized to one of the groups, and the tests needed to be carried out at the same time using similar imaging technology. Following the QUADAS-2, we assessed whether the gold standard of histopathology was performed in all patients and whether histopathological evaluation was applied to all of the lesions identified, whether by Lugol chromoendoscopy or only by NBI. We also determined whether all patients were included in the analysis.

### Summary measures and planned methods of analysis

In the meta-analysis, we calculated and showed sensitivity, specificity, and positive and negative likelihood values in forest plots. We also created summary receiver operating characteristic (sROC) curves and estimates of the areas under the curves. All of these variables were subjected to per-patient and per-lesion analyses. I-square was used to evaluate heterogeneity. Due to the high heterogeneity among the studies, the Dersimonian Laird random effects model was used for calculation. The sROC curves were created using the Moses-Littenberg linear model.

Meta-DiSc version 1.4 software was used (Unit of Clinical Biostatistics, Ramo e Cajal Hospital, Madrid, Spain).

## Results

### Study selection

In the searches conducted in the PubMed/Medline, LILACS, Cochrane CENTRAL, Scopus, and Cinahl databases, 7079 articles were identified. Of these, 7052 were excluded after evaluation of the title and abstract showed they were not related to the topic under study. Of the remaining 27 studies, seven were excluded for not using Lugol’s iodine solution in comparison with NBI [9–15] and one was excluded because it did not permit the diagnostic accuracy of the methods to be assessed. In this study, 25 endoscopists used a scale to assess the ease of identifying lesions in photos in each method [16].

The 19 remaining articles were assessed in full. One of these, in which the objective was to compare the tolerability of NBI to that of Lugol chromoendoscopy, was excluded because no patients with esophageal squamous cell carcinoma were evaluated using NBI [17].

Eighteen studies were included in the systematic review, but six were not included in the meta-analysis for the following reasons: Two studies considered low-grade dysplasias to be true positives, and it was not possible to separate and analyze the data from these studies, which consider them to be false positives [18, 19]. Two did not provide complete data to calculate sensitivity, specificity, and accuracy values in either the per-patient or per-lesion analysis [7, 20]. In one, the examinations were performed by inexperienced endoscopists [8]; and in another, the data were collected retrospectively [21].

The quantitative analysis was performed in twelve studies [22–33].

### Study characteristics

The twelve studies considered in the meta-analysis provided all the data necessary to compare the diagnostic accuracy of NBI and Lugol chromoendoscopy, whether in a per-patient or a per-lesion analysis, for a total of 1911 patients (Table 1). Of these, eleven studies were cross-sectional, and one was a prospective, randomized, controlled study. In the cross-sectional studies, both NBI and Lugol chromoendoscopy were performed sequentially in all patients-first NBI followed by Lugol chromoendoscopy in the same examination. In the randomized prospective study, the patients were separated into two homogeneous groups and each group was assessed using only one of the methods; the author compared the pink-color sign in iodine-negative areas of Lugol chromoendoscopy to capillary changes in the BAs in NBI [22].

**Table 1:** Studies characteristics

Study	Patients included in the analysis	Gold standard	Interval	Study design	Study inclusion criteria	Tests methods
Goda 2015	294	Histology	Two different groups	Prospective randomized	HNSCC/ESCC previous endoscopic resection	NBI x Lugol
Nagami 2014	202	Histology	Sequential approach	Cross-sectional	HNSCC/ESCC previous endoscopic resection	NBI x Lugol
Takahashi 2014	285	Histology	Sequential approach	Cross-sectional	HNSCC/ESCC	NBI x Lugol
Wang 2014	500	Histology	Sequential approach	Cross-sectional	HNSCC	NBI x Lugol
Ide 2013	43	Histology	Sequential approach	Cross-sectional	Achalasia	NBI x Lugol
Kawai 2012	103	Histology	Sequential approach	Cross-sectional	General population	NBI x Lugol
Ide 2011	129	Histology	Sequential approach	Cross-sectional	HNSCC	NBI x Lugol
Lecleire2011	30	Histology	Sequential approach	Cross-sectional	Cured ESCC without esophagectomy	NBI x Lugol
Takenaka 2009	142	Histology	Sequential approach	Cross-sectional	HNSCC	NBI x Lugol
Huang 2009	90	Histology	Sequential approach	Cross-sectional	Early ESCC or precancerous	NBI x Lugol
Lee 2009	44	Histology	Sequential approach	Cross-sectional	HNSCC	NBI x Lugol
Kuraoka 2009	49	Histology	Sequential approach	Cross-sectional	High risk patient for ESCC	NBI x Lugol
Total	1911					

The criteria for inclusion of the articles selected for the meta-analysis varied. Ten studies evaluated a high-risk population; of these, nine included patients with a history of squamous cell carcinoma of the head and neck region or the esophagus [22–25, 28–30, 32], one included patients with achalasia [26], and one did not specify [33]. One study evaluated the methods in patients who already had a diagnosis of pre-malignant lesions or squamous cell carcinoma of the esophagus [31]. The other study evaluated the methods in the general population [27].

The gold standard for all the studies was histopathological assessment of the lesions, but this analysis was only possible when at least one of the methods found any suspicious lesions. Patients without suspicious lesions as determined by the two methods were not subjected to histopathological evaluation and were considered true negatives. The criteria for obtaining the material for histopathological analysis also varied between the studies: any iodine-negative area and/or BAs, iodine-negative areas greater than or equal to 5 mm in size and/or BAs, or only iodine-negative areas greater than 5 mm. In these studies, BAs that stained with Lugol’s solution or Lugol voiding lesions smaller than 5 mm were not subjected to histopathological analysis were considered disease-free. No study conducted random biopsies on patients without suspicious areas.

The criteria to consider a lesion suspicious using NBI and Lugol chromoendoscopy were not consistent among the studies. All of the studies and the inclusion criteria of this review included BAs in NBI among the findings, whether they were associated with capillary changes or not. In Lugol chromoendoscopy, iodine-negative areas greater than or equal to 5 mm were included, regardless of pink-color sign.

### Risk of bias within studies (Table 2)

**Table 2:** Studies characteristics

			Goda 2015	Nagami 2014	Takahashi 2014	Wang 2014	Ide 2013	Kawai 2012	Ide 2011	Lecleire 2011	Takenaka 2009	Huang 2009	Lee 2009	Kuraoka 2009
PATIENT SELECTION	Signaling questions	Was a consecutive or random sample of patients enrolled?	Yes	Unclear	Unclear	Yes	Yes	Yes	Yes	Unclear	Unclear	Unclear	Yes	Unclear
		Was a case-control design avoided?	No	No	No	No	No	No	No	No	No	No	No	No
		Did the study avoided inappropriate exclusions?	No	No	No	No	No	Yes	No	No	No	No	No	Unclear
	Risk of bias	Could the selection of patients have introduced bias?	Low	Low	Low	Low	Low	Moderate	Low	Low	Low	Moderate	Low	Moderate
	Concerns regarding applicability	Are there concerns that the included patients do not match the review question?	Low	Low	Low	Low	Low	High	Low	Low	Low	High	Low	Low
INDEX TEST	Signaling questions	Were the index test results interpreted without knowledge of the results of the reference standard?	Yes	Yes	Yes	Unclear	Yes	Yes	Yes	Yes	Yes	No	Yes	Yes
		If a threshold was used, was it prespecified?	Yes	Yes	Yes	Yes	Yes	No	Yes	Yes	Yes	Yes	Yes	Yes
	Risk of bias	Could the conduct or interpretation of the index test have introduced bias?	Low	Low	Low	Low	Low	Moderate	Low	Low	Low	Moderate	Low	Low
	Concerns regarding applicability	Are there concerns that the index test, its conduct, or its interpretation differ from the review question?	High	Low	High	Low	Low	Unclear	Low	Low	Low	Low	Low	Low
REFERENCE STANDARD	Signaling questions	Is the reference standard likely to correctly classify the target condition?	Yes	Yes	Yes	Yes	Yes	No	Yes	Yes	Yes	No	Yes	Unclear
		Were the references standard results interpreted without knowledge of the results of the index test?	Yes	Yes	Yes	Unclear	Unclear	Unclear	Unclear	Yes	Yes	Unclear	Unclear	Unclear
	Risk of bias	Could the reference standard, its conduct, or its interpretation have introduced bias?	Low	Low	Low	Low	Low	High	Low	Low	Low	High	Low	Unclear
	Concerns regarding applicability	Are there concerns that the target condition as defined by the reference standard does not match the review question?	Low	Low	Low	Low	Low	High	Low	Low	Low	High	Low	Unclear
FLOW AND TIMING	Signaling questions	Was there an appropriate interval between index test(s) and reference standard?	Yes	Yes	Yes	Yes	Yes	Yes	Yes	Yes	Yes	Yes	Yes	Yes
		Did all patients receive a reference standard?	No	No	No	No	No	No	No	No	No	Unclear	No	Unclear
		Did all patients receive the same reference standard?	Yes	Yes	Yes	Yes	Yes	No	No	Yes	No	Yes	Yes	Yes
		Were all patients included in the analysis?	No	No	Yes	No	No	No	No	Yes	No	Yes	No	Unclear
	Risk of bias	Could the patient flow have introduced bias?	Moderate	Moderate	Low	Moderate	Moderate	High	Moderate	Low	High	Low	Moderate	Low

In assessing risk of bias in patient selection, we found that nine studies (75%) presented low risk and that the other three (25%) presented moderate risk. The risk index test was low in ten studies (83%) and moderate in two (17%). With regard to the gold standard, nine (75%) exhibited low risk; two (17%), high risk; and one (8%), undetermined. Flow and time risk was low in four studies (33%), moderate in six (50%), and high in two (17%).

The applicability of patient selection was high in ten studies (83%) and low in two (17%). The index test was high in nine studies (75%), low in two (17%), and undetermined in one (8%). The gold standard was high in nine studies (75%), low in two (17%), and undetermined in one (8%).

### Results of individual studies and Syntheses of results (see NBI and Lugol graphs)

**Fig. 1:**
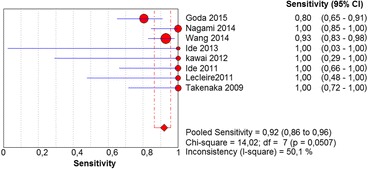
Forest plot: Lugol chromoendoscopy’s sensitivity per-patient analysis

**Fig. 2:**
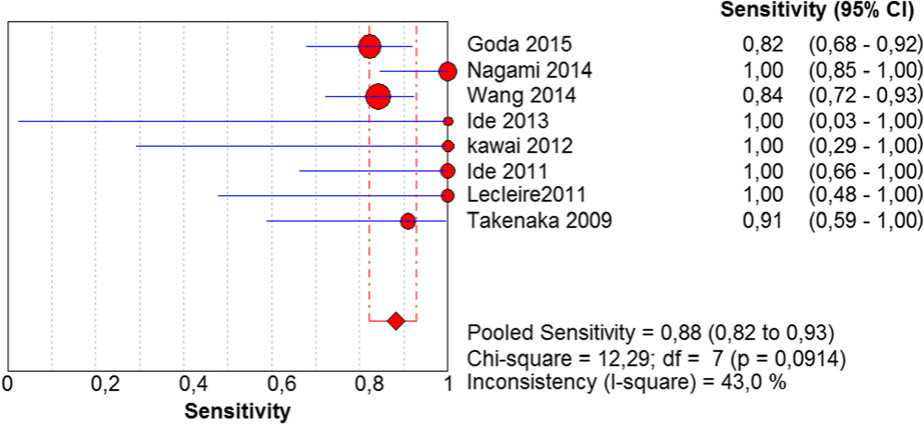
Forest plot: NBI’s sensitivity per-patient analysis

**Fig. 3:**
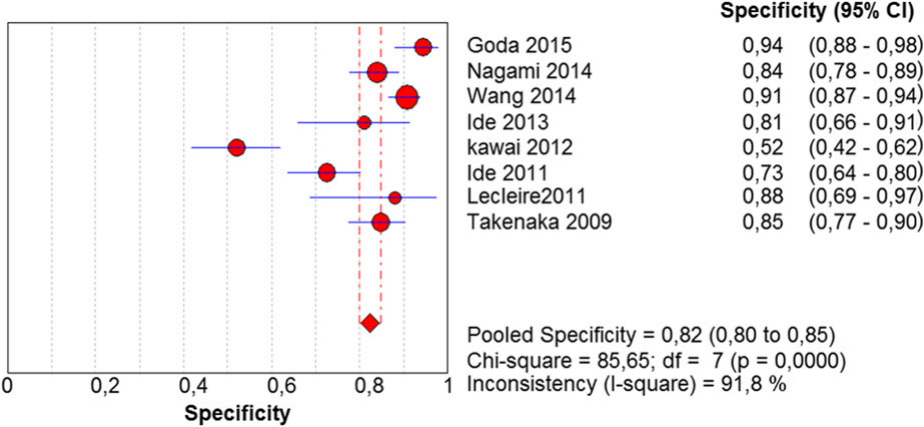
Forest plot: Lugol chromoendoscopy’s specificity per-patient analysis

**Fig. 4:**
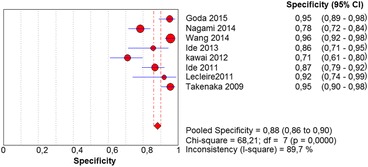
Forest plot: NBI’s specificity per-patient analysis

**Fig. 5:**
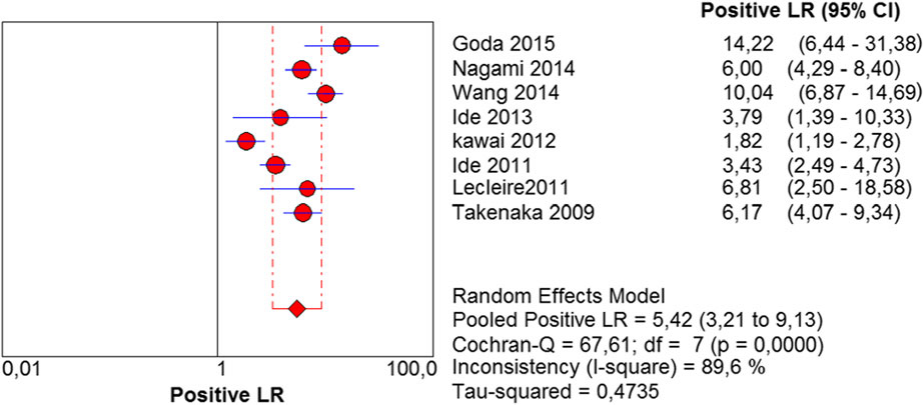
Forest plot: Lugol chromoendoscopy’s positive likelihood ratio per-patient analysis

**Fig. 6:**
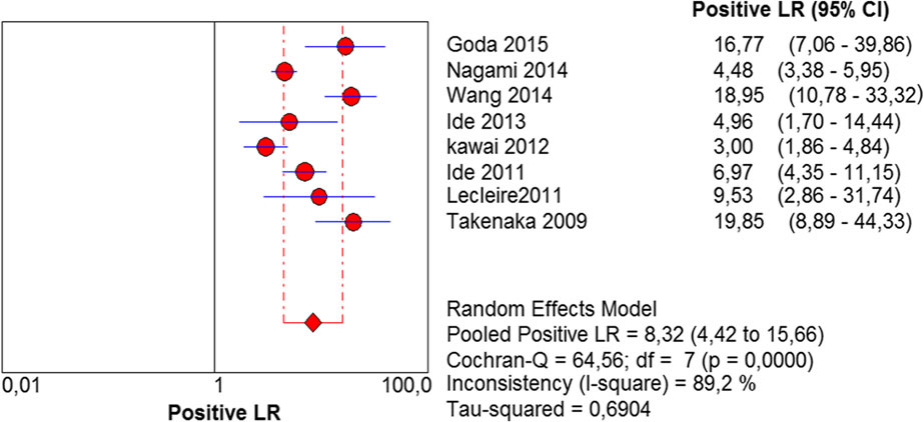
Forest plot: NBI’s positive likelihood ratio per-patient analysis

**Fig. 7:**
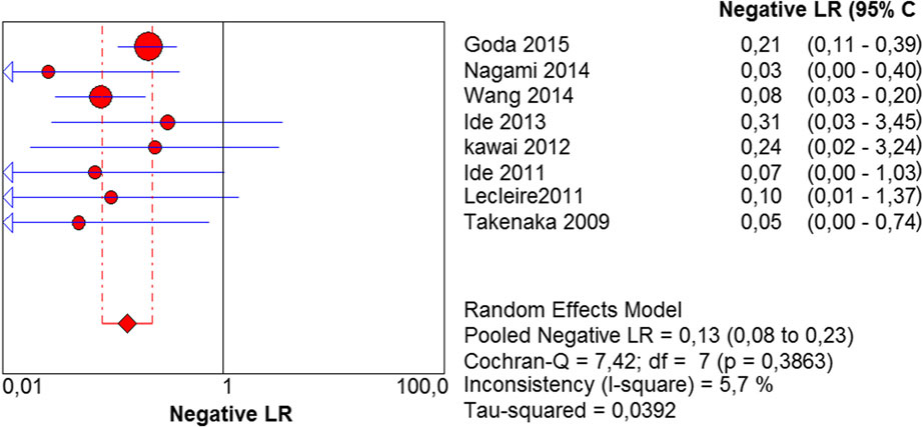
Forest plot: Lugol chromoendoscopy’s negative likelihood ratio per-patient analysis

**Fig. 8:**
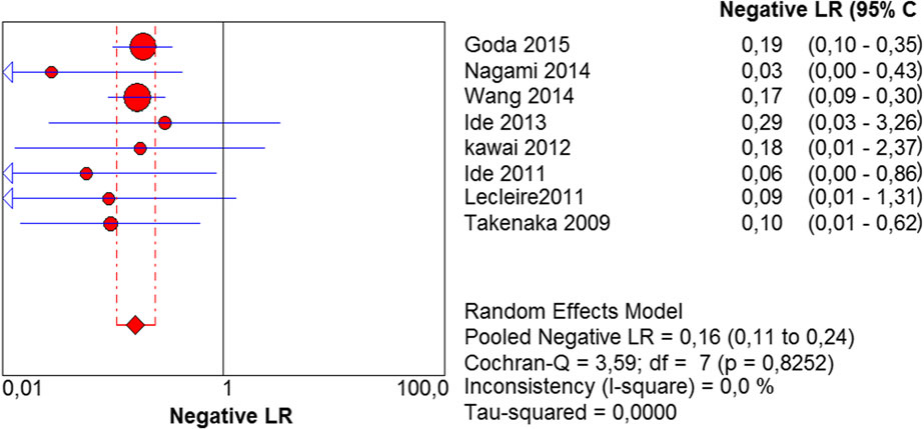
Forest plot: NBI’s negative likelihood ratio per-patient analysis

**Fig. 9:**
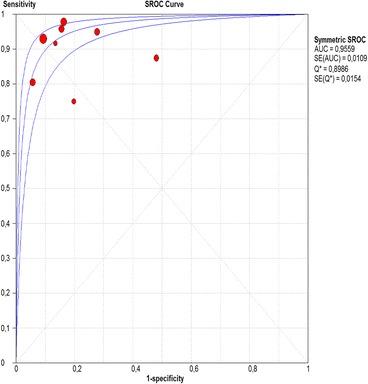
Summary receiver operating characteristic (sROC) curve for Lugol chromoendoscopy in per-patient analysis

**Fig. 10:**
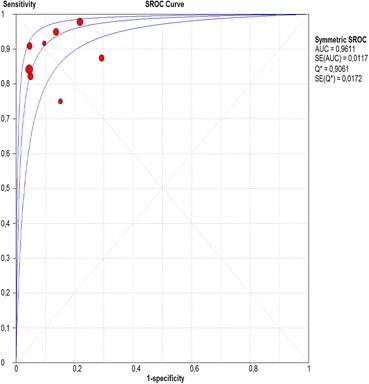
Summary receiver operating characteristic (sROC) curve for NBI in per-patient analysis

In the per-patient analysis, eight studies (1123 patients) were analyzed. Of these, high-grade dysplasia or squamous cell carcinoma of the esophagus was diagnosed in 149 patients (13.26%). Sensitivity of Lugol chromoendoscopy was 92% (95% confidence interval (CI), 86 to 96% and heterogeneity, 50.1%) (Fig. 1). Sensitivity of NBI was 88% (95% CI, 86 to 93% and heterogeneity, 43%) (Fig. 2), and there was no significant difference between the methods. Specificity of Lugol chromoendoscopy was 82% (95% CI, 80 to 85% and heterogeneity, 91.8%) (Fig. 3). Specificity of NBI was 88% (95% CI, 86 to 90% and heterogeneity, 89.7%) (Fig. 4). These results demonstrate that NBI specificity is significantly superior to that of Lugol chromoendoscopy. Positive likelihood ratio for Lugol chromoendoscopy was 5.42 (95% CI, 3.21 to 9.13 and heterogeneity, 89.6%) (Fig. 5) and for NBI was 8.32 (95% CI, 4.42 to 15.66 and heterogeneity, 89.2%) (Fig. 6); there was no statistically significant difference between the methods. Negative likelihood ratio for Lugol chromoendoscopy was 0.13 (95% CI, 0.08 to 0.23 and heterogeneity, 5.7%) (Fig. 7) and for NBI was 0.16 (95% CI, 0.11 to 0.24 and heterogeneity, 0%) (Fig. 8), and there was no statistically significant difference between the methods. The post-test probability of Lugol chromoendoscopy in the studied population was 0.44; the probability of NBI was 0.54. The area under the sROC curve for Lugol chromoendoscopy was 0.9559 (Fig. 9); for NBI, this value was 0.9611 (Fig. 10).

**Fig. 11:**
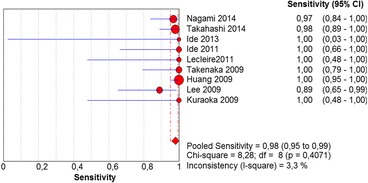
Forest plot: Lugol chromoendoscopy’s sensitivity per-lesion analysis

**Fig. 12:**
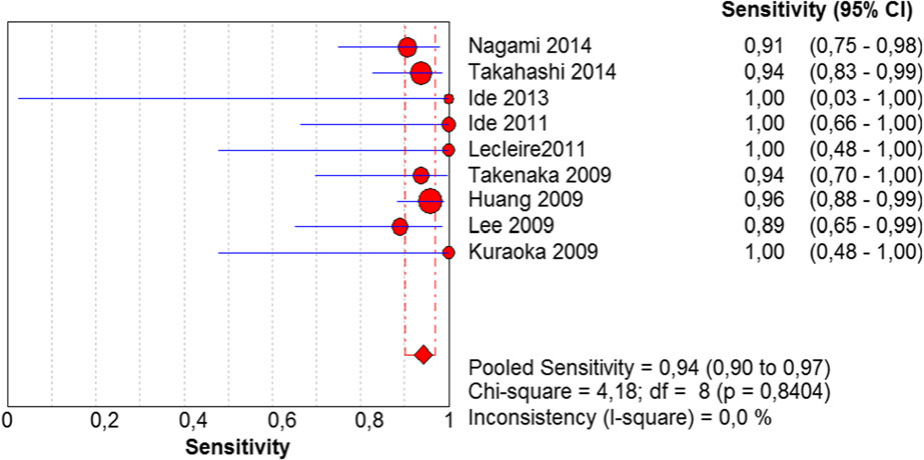
Forest plot: NBI’s sensitivity per-lesion analysis

**Fig. 13:**
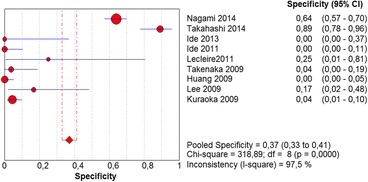
Forest plot: Lugol chromoendoscopy’s specificity per-lesion analysis

**Fig. 14:**
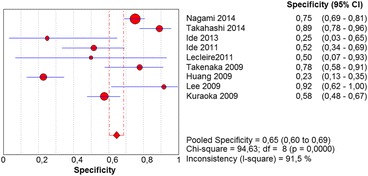
Forest plot: NBI’s specificity per-lesion analysis

**Fig. 15:**
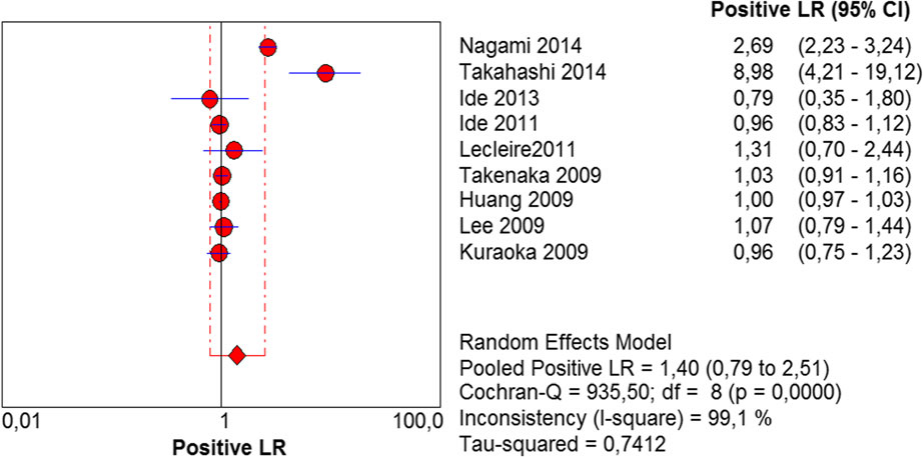
Forest plot: Lugol chromoendoscopy’s positive likelihood ratio per-lesion analysis

**Fig. 16:**
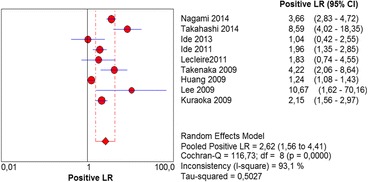
Forest plot: NBI’s positive likelihood ratio per-lesion analysis

**Fig. 17:**
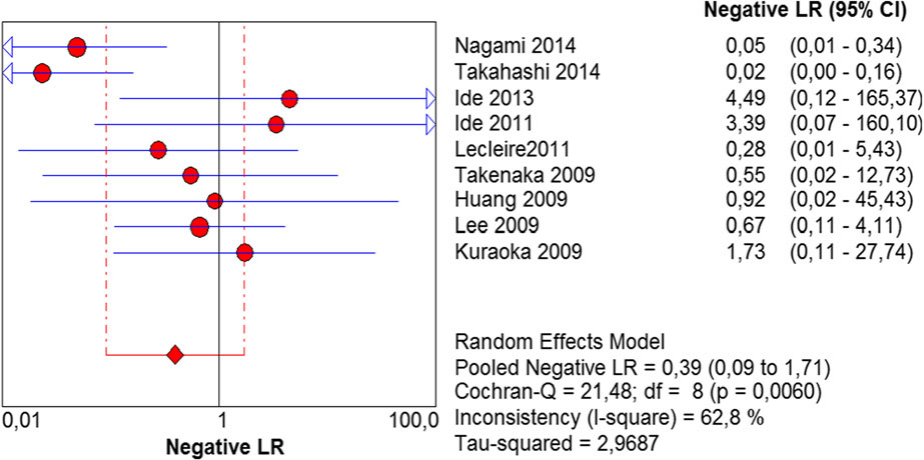
Forest plot: Lugol chromoendoscopy’s negative likelihood ratio per-lesion analysis

**Fig. 18:**
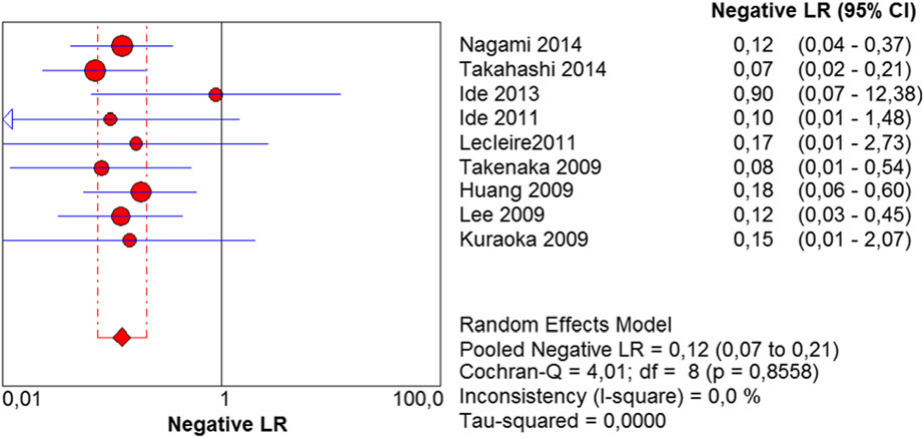
Forest plot: NBI’s negative likelihood ratio per-lesion analysis

**Fig. 19:**
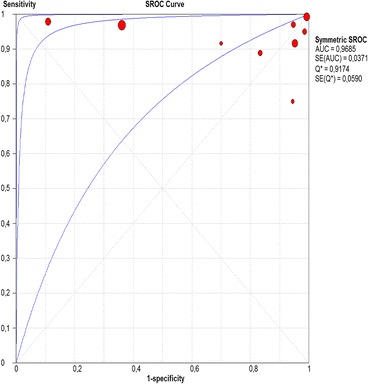
Summary receiver operating characteristic (sROC) curve for Lugol chromoendoscopy in per-lesion analysis

**Fig. 20:**
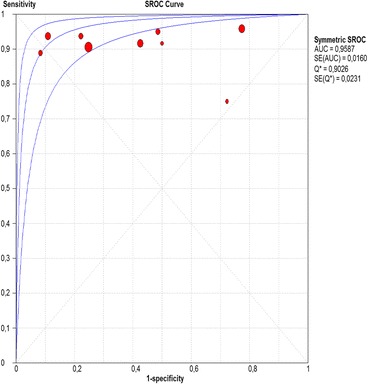
Summary receiver operating characteristic (sROC) curve for NBI in per-lesion analysis

The per-lesion analysis considered nine articles with a total of 746 lesions. Of these, 206 (27.61%) were high-grade dysplasias or squamous cell carcinomas of the esophagus. Sensitivity of Lugol chromoendoscopy was 98% (95% CI, 95 to 99% and heterogeneity, 3.3%) (Fig. 11). NBI sensitivity was 94%, (95% CI, 90 to 97% and heterogeneity, 0%) (Fig. 12), and there was no statistically significant difference between the methods. Specificity of Lugol chromoendoscopy was 37%, (95% CI, 33 to 41% and heterogeneity, 97.5%) (Fig. 13). NBI specificity was 65%, (95% CI, 60 to 69% and heterogeneity, 91.5%) (Fig. 14). These results demonstrate that NBI specificity is superior to that of Lugol chromoendoscopy, with statistical significance. Positive likelihood ratio for Lugol chromoendoscopy was 1.4, (95% CI, 0.79 to 2.51 and heterogeneity, 99.1%) (Fig. 15). In the case of NBI, this value was 2.62 (95% CI, 1.56 to 4.41 and heterogeneity, 93.1%) (Fig. 16), and there was no statistically significant difference between the methods. Negative likelihood ratio for Lugol chromoendoscopy was 0.39, (95% CI, 0.09 to 1.71 and heterogeneity, 62.8%) (Fig. 17) and for NBI was 0.12 (95% CI, 0.07 to 0.21 and heterogeneity, 0%) (Fig. 18), and there was no statistically significant difference between the methods. The post-test probability of Lugol chromoendoscopy for the lesions identified in the studied population was 0.37, while the same probability of NBI was 0.50. The area under the sROC curve in the case of Lugol chromoendoscopy was 0.9685 (Fig. 19); in the case of NBI, this value was 0.9587 (Fig. 20).

## Discussion

### Summary of evidence

Considering the need to identify subtle changes in the esophageal mucosa for the diagnosis of squamous cell carcinoma in early stages with healing potential, various methods are being studied. Traditionally, esophageal chromoscopy with Lugol’s iodine solution is used in high-risk patients to increase the rate of detection for these lesions relative to white light esophagoscopy [5, 6]. Because of the risks of complications inherent to the use of this dye and because its application is not practical, other methods have been developed and compared. One of these methods is NBI, which is desirable because of its convenience and because it eliminates the risks inherent to the use of Lugol’s solution while providing comparable diagnostic accuracy.

The meta-analysis conducted in this systematic review showed no statistically significant differences between the sensitivity of Lugol chromoendoscopy and that of NBI for diagnosing high-grade dysplasia and squamous cell carcinoma of the esophagus in a per-patient or per-lesion analysis. These sensitivities were as follows: Lugol chromoendoscopy per-patient and per-lesion: 92 and 88%, respectively, and NBI per-patient and per-lesion: 98 and 94%, respectively. Most of the published papers came to the same conclusion, except for two [18, 19]. These two included low-grade dysplasia in the true positives, a finding that was regarded as a methodological error in this review and consequently excluded from the meta-analysis.

As the articles showed, specificities of NBI were superior to those of Lugol chromoendoscopy with statistical significance, both in per-patient and a per-lesion analysis. These specificities were as follows: NBI per-patient and per-lesion: 88 and 82%, respectively, and Lugol chromoendoscopy per-patient and per-lesion: 65 and 37%, respectively.

Considering the calculations of this meta-analysis, the positive and negative likelihood values for Lugol chromoendoscopy in per-lesion analysis failed to obtain statistical significance. This is because its CI includes the number 1, which corresponds to the neutral number.

In the per-patient analysis, the positive likelihood value of Lugol chromoendoscopy was 5.42. This finding demonstrates that Lugol chromoendoscopy on a suspicious area increased the 13.26% risk in the studied population to 45%. The positive likelihood value for NBI was 8.32, increasing the risk of this population to 56% when the test was positive. There was no statistically significant difference between the methods. Similarly, the post-test probability calculation showed that the risk of high-grade dysplasia or squamous cell carcinoma of the esophagus in patients with suspicious areas detected in Lugol chromoendoscopy in this population was 44%, while in chromoendoscopy with NBI, this risk was 54%. The negative likelihood value of Lugol chromoendoscopy was 0.13, indicating that patients without suspicious lesions in Lugol chromoendoscopy have their initial disease risk reduced to 1.9%. The value for negative likelihood for NBI was 0.16, taking initial risk reduction to 2.3%. There was no statistically significant difference between the methods.

In the per-lesion analysis, the positive and negative likelihood values for Lugol chromoendoscopy were 2.62 and 0.12, respectively. This finding demonstrated that for each positive lesion in NBI, the initial risk of 27.61% for lesions in general rises to 50%, and for each negative lesion, this risk drops to 4%. The post-test probability calculation showed that the risk of high-grade dysplasia or squamous cell carcinoma for suspicious areas in Lugol chromoendoscopy in this population was 37%, while in chromoendoscopy with NBI, this risk was 50%.

The areas under the sROC curve in the per-patient analysis were 0.9559 for Lugol chromoendoscopy and 0.9611 for NBI, demonstrating good accuracy of the methods with no significant difference between them. In the per-lesion analysis, these areas were 0.9685 for Lugol chromoendoscopy and 0.9587 for NBI. However, the CI in the sROC curve for Lugol chromoendoscopy was found to be very extensive, a finding that suggests low credibility. NBI also demonstrated good accuracy in the per-lesion analysis.

### Limitations

The fact that there is no gold standard applicable to all patients is a limitation of the articles included in this systematic review, as is the fact that endoscopy is an operator-dependent examination. Histopathological evaluation depended on the identification of lesions by NBI or Lugol chromoendoscopy. In this context, patients without suspicious lesions identified by either method were considered not to have the illness and were there not evaluated using the gold standard. The criteria for histopathological examination of suspicious lesions varied between the studies; some regarded the sensitivity of Lugol chromoendoscopy as being so high that patients without suspicious lesions in Lugol chromoendoscopy were considered not to have the disease regardless of the NBI findings, even without histopathological evaluation.

In general, the methodology of the studies favored the sensitivity of Lugol chromoendoscopy if it was performed by the same endoscopist shortly after esophageal evaluation with NBI. Additionally, the articles that did not submit lesions that were positive NBI but not suspicious in Lugol chromoendoscopy to the gold standard began with 100% sensitivity for Lugol chromoendoscopy.

## Conclusions

According to both forms of analysis, NBI presented the same rate of detection of high-grade dysplasia and esophageal squamous cell carcinoma when compared to Lugol. Regarding the differentiation of high-grade dysplasia and squamous cell carcinoma from other esophageal mucosa alterations, NBI was shown to be superior to Lugol. Thus, we can conclude that NBI was adequate in evaluating the esophagus in order to diagnose high-grade dysplasia and squamous cell carcinoma.

## Abbreviations

BA: Brownish area
CI: Confidence Interval
ESCC: Esophageal squamous cell carcinoma
HNSCC: Head and neck squamous cell carcinoma
NBI: Narrow Band Imaging
sROC: Summary Receiver Operating Characteristic

## References

[1] GLOBOCAN (2012). Estimated Cancer Incidence, Mortality and Prevalence Worldwide in 2012. International Agency for Research on Cancer-World Health Organization.

[2] Zhag Y (2013). Epidemiology of esophageal cancer. World J Gastroenterol.

[3] Torre LA, Bray F, Siegel RL (2015). Global cancer statistics, 2012. CA Cancer J Clin.

[4] Gibson MK, Tanabe KK, Goldberg RM, Savarese DMF (2015). Epidemiology, pathobiology, and clinical manifestations of esophageal cancer. UpToDate Dec.

[5] Carvalho R, Areia M, Brito D, Saraiva S, Alves S, Cadime AT (2013). Diagnostic accuracy of lugol chromoendoscopy in the esophagus in patients with head and neck cancer. Rev Esp Enferm Dig.

[6] Hashimoto CL, Iriya K, Baba ER, Navarro-Rodriguez T, Zerbini MC, Eisiq JN, Barbuti R, Chinzon D, Moraes-Filho JP (2005). Lugol’s dye spray chromoendoscopy establishes early diagnosis of esophageal cancer in patients with primary head and neck cancer. Am J Gastroenterol.

[7] Ishihara R, Takeuchi Y, Chatani R, Kidu T, Inoue T, Hanaoka N, Yamamoto S, Higashino K, Uedo N, Iishi H, Tatsuta M, Tomita Y, Ishiguro S (2010). Prospective evaluation of narrow-band imaging endoscopy for screening of esophageal squamous mucosal high-grade neoplasia in experienced and less experienced endoscopists. Dis Esophagus.

[8] Yokoyama A, Ichimasa K, Ishiguro T, Mori Y, Ikeda H, Hayashi T, Minami H, Hayashi S, Watanabe G, Inoue H, Kudo S (2012). Is it proper to use non-magnified narrow-band imaging for esophageal neoplasia screening? Japanese single-center, prospective study. Dig Endosc.

[9] Chai TH, Jin XF, Li SH, Du RL, Zhang J (2014). A tandem trial of HD-NBI versus HD-WL to compare neoplasia miss rates in esophageal squamous cell carcinoma. Hepatogastroenterology.

[10] Nagai K, Ishihara R, Ishiguro S, Ohta T, Kanzaki H, Yamashina T, Aoi K, Matsuura N, Ito T, Fuji M, Yamamoto S, Hanaoka N, Takeuchi Y, Higashino K, Uedo N, Iishi H, Tatsuta M, Tomita Y, Matsunaga T (2014). Endoscopic optical diagnosis provides high diagnostic accuracy of esophageal squamous cell carcinoma. BMC Gastroenterol.

[11] Minami H, Inoue H, Ikeda H, Satodate H, Hamatani S, Nakao K, Kudo SE (2012). Usefulness of background coloration in detection of esophago-pharyngeal lesions using NBI magnification. Gastroenterol Res Pract.

[12] Wang SF, Yang YS, Yuan J, Zhang XL, Lu ZS, Sun G, Linghu EQ, Meng JY (2012). The value of esophageal intrapapillary capillary loop visualized by magnifying narrow-band imaging endoscopy in diagnosing esophageal mucosal pathology. Zhonghua Nei Za Zhi.

[13] Ishihara R, Inoue T, Uedo N, Yamamoto S, Kawada N, Tsujii Y, Kanzaki H, Hanafusa M, Hanaoka N, Takeuchi Y, Higashino K, Iishi H, Tatsuta M, Tomita Y, Ishiguro S (2010). Significance of each narrow-band imaging finding in diagnosing squamous mucosal high-grade neoplasia of the esophagus. J Gastroenterol Hepatol.

[14] Muto M, Minashi K, Yano T, Saito Y, Oda I, Nonaka S, Omori T, Sugiura H, Goda K, Kaise M, Inoue H, Ishikawa H, Ochiai A, Watanabe H, Tajiri H, Saito D (2010). Early detection of superficial squamous cell carcinoma in the head and neck region and esophagus by narrow band imaging: a multicenter randomized controlled trial. J Clin Oncol.

[15] Lee CT, Chang CY, Lee YC, Tai CM, Wang WL, Tseng PH, Hwang JC, Hwang TZ, Wang CC, Lin JT (2010). Narrow-band imaging with magnifying endoscopy for the screening of esophageal cancer in patients with primary head and neck cancers. Endoscopy.

[16] Yoshida Y, Goda K, Tajiri H, Urashima M, Yoshimura N, Kato T (2009). Assessment of novel endoscopic techniques for visualizing superficial esophageal squamous cell carcinoma: autofluorescence and narrow-band imaging. Dis Esophagus.

[17] Yamasaki Y, Takenaka R, Hori K, Takemoto K, Kawano S, Kawahara Y, Okada H, Fujiki S, Yamamoto K (2015). Tolerability of magnifying narrow band imaging endoscopy for esophageal cancer screening. World J Gastroenterol.

[18] Chung CS, Liao LJ, Lo WC, Chou YH, Chang YC, Lin YC, Hsu WF, Shueng PW, Lee TH (2013). Risk factors for second primary neoplasia of esophagus in newly diagnosed head and neck cancer patients: a case-control study. BMC Gastroenterol.

[19] Kim do H, Gong EJ, Jung HY, Lim H, Ahn JY, Choi KS, Lee JH, Choi KD, Song HJ, Lee GH, Kim JH, Roh JL, Nam SY, Kim SY, Baek S (2014). Clinical significance of intensive endoscopic screening for synchronous esophageal neoplasm in patients with head and neck squamous cell carcinoma. Scand J Gastroenterol.

[20] Gong EJ, Kim DH, Ahn JY, Choi KS, Jung KW, Lee JH, Choi KD, Song HJ, Lee GH, Jung HY, Kim JH, Roh JL, Choi SH, Nam SY, Kim SY (2015). Routine endoscopic screening for synchronous esophageal neoplasm in patients with head and neck squamous cell carcinoma: a prospective study. Dis Esophagus.

[21] Asada-Hirayama I, Kodashima S, Fujishiro M, Ono S, Niimi K, Mochizuki S, Konno-Shimizu M, Mikami-Matsuda R, Minatsuki C, Nakayama C, Takahashi Y, Yamamichi N, Koike K (2013). Narrow band imaging with magnification can pick up esophageal squamous cell carcinoma more efficiently than lugol chromoendoscopy in patients after chemoradiotherapy. Diagn Ther Endosc.

[22] Goda K, Dobashi A, Yoshimura N, Kato M, Aihara H, Sumiyama K, Toyoizumi H, Kato T, Ikegami M, Taijiri H (2015). Narrow-Band Imaging Magnifyin Endoscopy versus Lugol Chromoendoscopy with Pink-Color Sign Assessment in the Diagnosis of Superficial Esophageal Squamous Neoplasms: A Randomised Noninferiority Trial. Gastroenterol Res Pract.

[23] Nagami Y, Tominaga K, Machida H, Nakatani M, Kameda N, Sugimori S, Okazaki H, Tanigawa T, Yamagami H, Kudo N, Shiba M, Watanabe K, Watanabe T, Iguchi H, Fujiwara Y, Ohira M, Hirakawa K, Arakawa T (2014). Usefulness of non-magnifying narrow-band imaging in screening of early esophageal squamous cell carcinoma: a prospective comparative study using propensity score matching. Am J Gastroenterol.

[24] Takahashi M, Shimizu Y, Ono M, Suzuki M, Omori S, Yoshida T, Mori Y, Nakagawa M, Ono S, Nakagawa S, Mabe K, Kato M, Hatanaka K, Asaka M, Sakamoto N (2014). Endoscopic diagnosis of early neoplasia of the esophagus with narrow band imaging: correlations among background coloration and iodine staining findings. J Gastroenterol Hepatol.

[25] Wang CH, Lee YC, Wang CP, Chen CC, Ko JY, Han ML, Chen TC, Lou PJ, Yang TL, Hsiao TY, Wu MS, Wang HP, Tseng PH (2014). Use of transnasal endoscopy for screening of esophageal squamous cell carcinoma in high-risk patients: yield rate, completion rate, and safety. Dig Endosc.

[26] Ide E, Carneiro FOAA, Frazão MSV, Chaves DM, Sallum RAA, de Moura EGH, Sakai P, Maluf-Filho F (2013). Endoscopic Detection of Early Esophageal Squamous Cell Carcinoma in Patients with Achalasia: Narrow-Band Imaging versus Lugol’s Staining. J of Oncology.

[27] Kawai T, Takagi Y, Yamamoto K, Hayama Y, Fukuzawa M, Yagi K, Fukuzawa M, Kataoka M, Kawakami K, Itoi T, Moriyasu F, Matsubabayashi J, Nagao T (2012). Narrow-band imaging on screening of esophageal lesions using an ultrathin transnasal endoscopy. J Gastroenterol Hepatol.

[28] Ide E, Maluf-Filho F, Chaves DM, Matuguma SE, Sakai P (2011). Narrow-band imaging without magnification for detectinh early esophageal squamous cell carcinoma. World J Gastroenterol.

[29] Lecleire S, Antonietti M, Iwanicki-Caron I, Duclos A, Lemoine F, Pessot FL, Michel P, Ducrotté P, Fiore F (2011). Lugol chromo-endoscpy versus narrow band imaging for endoscopic screening of esophageal squamous-cell carcinoma in patients with a history of cured esophageal cancer: a feasibility study. Dis Esophagus.

[30] Takenaka R, Kawahara Y, Okada H, Hori K, Inoue M, Kawano S, Tanioka D, Tsuzuki T, Uemura M, Ohara N, Tominaga S, Onoda T, Yamamoto K (2009). Narrow-band imaging provides reliable screening for esophageal malignancy in patients with head and neck cancers. Am J Gastroenterol.

[31] Huang LY, Cui J, Wu CR, Liu YX, Xu N (2009). Narrow-band imaging in the diagnosis of early esophageal cancer and precancerous lesions. Chin Med J (Engl).

[32] Lee YC, Wang CP, Chen CC, Chiu HM, Ko JY, Lou PJ, Yang TL, Huang HY, Wu MS, Lin JT, Hsiu-Hsi Chen T, Wang HP (2009). Transnasal endoscopy with narrow-band imaging and Lugol staining to screen patients with head and neck câncer whose condition limits oral intubation with standard endoscope (with vídeo). Gastrointest Endosc.

[33] Kuraoka K, Hoshino E, Tsuchida T, Fujisaki J, Takahashi H, Fujita R (2009). Early esophageal cancer can be detected by screening endoscopy assisted with narrow-band imaging (NBI). Hepatogastroenterology.

